# Operative hysteroscopy with the Bigatti shaver (IBS ®) for the removal of placental remnants

**Published:** 2018-09

**Authors:** SH Ansari, G Bigatti, MM Aghssa

**Affiliations:** Department of Obstetrics and Gynecology, Day General Hospital, Valiasr St, Tehran, Iran; Sino European Life Expert Centre, Shanghai Jiaotong University Affiliated Renji Hospital.160 Pujian Rd. Shanghai, China; Department of Obstetrics and Gynecology, Day General Hospital, Valiasr St, Tehran, Iran.

**Keywords:** Placental remnants, Operative hysteroscopy, Intrauterine Bigatti Shaver, Retained product of conception

## Abstract

**Background:**

About 15-20% of pregnant women will miscarry spontaneously during the first trimester. Traditionally, the surgical treatment of placental remnants has been dilation and curettage (D&C). However, because of its ‘blind’ nature there is a risk of serious complications, such as infection, adhesion, uterine perforation, or bowel injury. Hysteroscopy, with direct visualization of the uterine cavity, decreases these complications. In this retrospective case series we evaluated the efficacy and the feasibility of operative hysteroscopy using the Intrauterine Bigatti Shaver (IBS ® ) for the treatment of placental remnants, both, in a University hospital in Italy and in a private hospital in Iran.

**Materials and Methods:**

From December 2013 to April 2018 a retrospective medical records review, of patients undergoing placental remnant removal with the IBS ® , was performed. Sixty-five patients suspected of retained products of conception (RPOC) underwent operative hysteroscopy during this period using the IBS ® . Placental remnants were confirmed histologically in 52 cases (80%). The median age of the patients was 36 years. Placental remnants were observed after 42 early miscarriages, 5 terminations of pregnancy, 4 vaginal deliveries and 1 cesarean delivery. Thirty-two patients had abnormal uterine bleeding, 15 were asymptomatic and 5 had subfertility after miscarriage. Most cases (90%) were diagnosed by transvaginal ultrasound.

**Results:**

The median interval between surgery and the end of pregnancy was 56 days (a range of 15-90 days). The size of placental remnants was between 15 and 30mm. Three women showed a cavity filled with placental tissue residual (more than 4cm). The median resection time was 4.5 minutes and the median total surgery time was 6.6 minutes. Median fluid deficit [saline solution] was 240 ml. In two cases there was excessive intraoperative bleeding, and one patient required a conversion to bipolar resectoscope for hemostatic reasons. No perforation or postoperative complications occurred. There was no need for second-look operative hysteroscopy and postoperative ultrasound confirmed complete evacuation of the RPOC. Only one patient had a minor adhesion.

**Conclusion:**

The IBS ® seems to be an effective and safe instrument for the removal of placental remnants. It allows for short operation time with a high success and low complication rate.

## Introduction

About 15-20% of pregnant women will miscarry spontaneously during the first trimester (Luise et al., 2002). Although first trimester miscarriages can be successfully managed conservatively, 20% of patients do not respond to expectant and conservative management after 4 weeks. In these cases, surgical treatment is recommended (Luise et al., 2002; Zhang et al., 2005). Patients with miscarriage could experience placental remnants with symptoms including vaginal bleeding (80%), fever (5%) and abdominal or pelvic pain (5%) (Maslovitz et al., 2004). However, as some cases of placental remnants may be asymptomatic, ultrasound is recommended for more accurate diagnosis. Ultrasound is a reliable method to predict whether placental remnants will evacuate spontaneously or require intervention (Abbasi et al., 2008).

Traditionally, the surgical treatment of placental remnants is dilation and curettage (D&C) which has been the most widely used treatment for the removal of products of conception (RPOC) (Pather et al., 2005). However, there is a high risk of serious complications, such as infection, adhesion, uterine perforation, or bowel injury.

Furthermore, it is well known that ‘blind’ procedures, such as too aggressive curettage, may damage the basal layer of the endometrium, leading to intrauterine adhesion formation (Asherman Syndrome) (Westendorp et al., 1998; Hooker et al., 2011). Some studies have reported intra uterine adhesions (IUAs) in 20% of women following surgical treatment for RPOC.

D&C is associated with more IUAs and incomplete removal, compared to operative hysteroscopy (Hooker et al., 2016). Therefore, products of conception removal under direct vision should be the treatment of choice. In such cases hysteroscopy was found to be complementary to ‘blind’ curettage in identifying and removing intrauterine lesions (Goldenberg et al., 1997). Hysteroscopy has been described in cases of postabortal or postpartum bleeding treatment, 3 decades ago (Tjanina et al., 2013). Hysteroscopy confirmed its value as a safe and precise procedure for the removal of placental remnants (Golan et al., 2011).

The Intrauterine Bigatti Shaver, (IBS ® ), has been able to selectively remove products of conception under direct visual control. The advantages of this technique include a small 8.5mm sheath diameter and its speed, with minimal injury and damage to the healthy endometrium. In addition, the Shaver technique did not cause any thermal injury of the uterine cavity. This may lead to reduced postoperative adhesion formation. IBS® has already proved to be a safe and efficient technique for endometrial polyps’ removal (Bigatti, 2011; Bigatti et al., 2012).

The aim of this study is to evaluate whether the IBS ® technique should be considered as an alternative approach for the removal of placental remnants on the basis of safety and success rate.

## Materials and Methods

### Patients

From December 2013 to April 2018, 65 medical records of patients treated for removal of placental remnants with the Shaver technique were evaluated. All patients were suspected of having RPOCs. Histology confirmed placental remnants in 52 patients (80%) after surgery. These patients underwent IBS ® removal of placental remnants in two different centers: Milan (Italy) and Tehran (Iran). Clinical records of each patient were collected and reviewed. The median age was 36 years, 22 patients were nulliparous, 23 patients were primiparous, and 7 patients were secondiparous. Patients’ Surgical details are shown in Table I.

**Table I:** Patients’ clinical characteristics.

Age, years	36 (25-42)
Parity	1 (0-2)
Previous uterine surgery, n (%)	21 (40%) None 14 (27%) D&C for miscarriage <10 weeks 10 (19%) Cesarean section 6 (12%) TOP for chromosomal or severe anatomical malformation >12 weeks 1 (2%) Manual removal of placenta after vaginal delivery

D&C = dilation and curettage; TOP = termination of pregnancy

**Table II:** Patients’ obstetric characteristics.

Obstetric event leading to placental remnants, n (%)	42 (80%) Miscarriage <10 weeks 5 (10%) TOP 4 (8%) Vaginal delivery (one had D&C for PPH) 1 (2%) Cesarean section
Symptoms, n (%)	32 (61%) Abnormal uterine bleeding 15 (29%) Asymptomatic 5 (10%) Amenorrhea and subfertility
Method of diagnosis of placental remnants, n (%)	47 (90%) Transvaginal ultrasound 2 (4%) Outpatient hysteroscopy 2 (4%) Transvaginal ultrasound + diagnostic hysteroscopy 1 (2%) Saline solution sonography

D&C = dilation and curettage; TOP = termination of pregnancy; PPH = postpartum hemorrhage

### Diagnosis and distribution of patients

Most cases were diagnosed with transvaginal ultrasound. If a previous uterine surgery has been held, it was reported as risk factor for abnormal placentation in 31 patients (66%): 14 patients had D&C at less than 10 weeks of gestation miscarriage, and 10 had previous cesarean sections. Six patients had termination of pregnancy (TOP) for chromosomal abnormality at >12 weeks, and 1 had a manual removal of placenta after vaginal delivery. Placental remnants occurred mainly after early miscarriage (<10 weeks) in 42 patients (80%), in 5 others (10 %) after termination of pregnancy (TOP, in 4 cases after vaginal deliveries (8%), and in 1 case after cesarean section in a twin pregnancy (2%).

Patients were referred to our clinics with different symptoms: 32 patients had abnormal uterine bleeding (61%), 15 were asymptomatic (29%), and 5 had been affected by subfertility and amenorrhea (10%) lasting more than 2 months after miscarriage. If there was a suspicion of a RPOC, different diagnostic approaches were performed.

Patients underwent surgery at a median of 56 days (range: 15-90 days) after the obstetrical event (Table III).

**Table III:** Surgery outcomes.

Time since end of pregnancy, median (range), days		56 (15-90)
Duration of surgery, median (range), minutes		
	Hysteroscopic resection	4.5 (3-8)
	Total procedure time	6.6 (5-10)
Fluid balance, median (range), mL		
	Inflow	1380 (700-4000)
	Outflow	1200 (500-3500)
	Deficit	240 (100-400)

### Surgical procedure

Surgeons with different levels of experience performed all the operations under general anesthesia. Cervical dilatation was done with a Hegar dilator up to No. 8. Normal saline (NaCl 0.9%) was used as distention media.

In all patients the IBS ® , Intrauterine Bigatti Shaver (Karl Storz GmbH & Co. of Tuttlingen) was used.

This device used a mechanical rotational cutting power with no electrical current. Tissue chips were removed at the same time as resection. A full and detailed description of the Shaver technique, including equipment and indications, has been well described in a recent publication (Bigatt, 2018).

## Results

**Figure 1:**
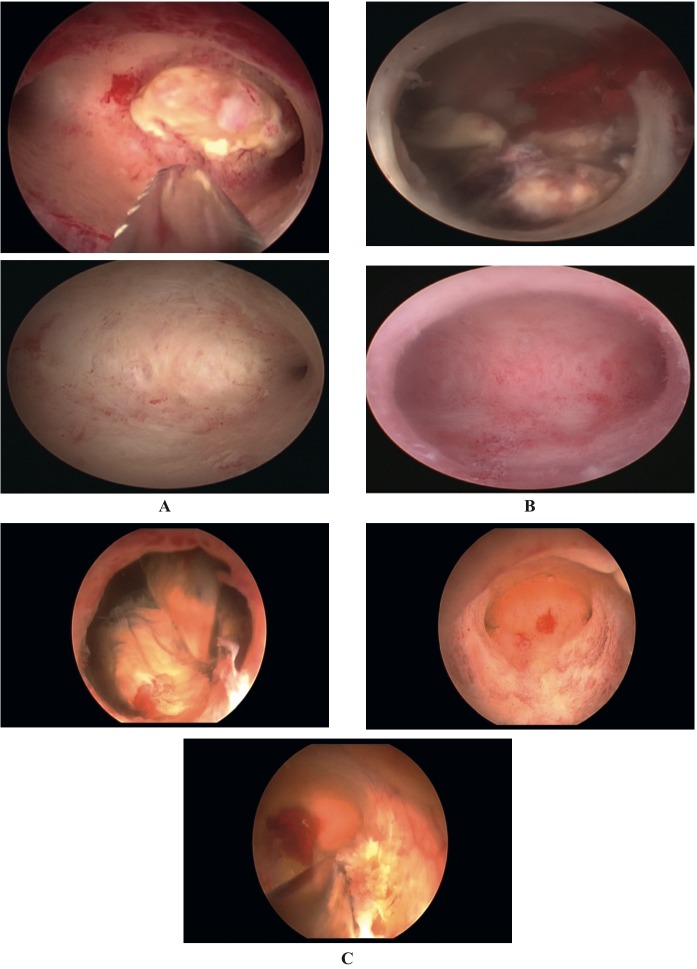
— Placental remnant before and after treatment with the Intrauterine Bigatti Shaver. A. 42-year-old nulliparous woman with a placental remnant of 15 mm after a miscarriage at <10 weeks; patient was asymptomatic, and an ultrasound scan revealed a retained product of conception. B. 42-year-old pluriparous woman with a placental remnant of 20 mm after an abortion in a twin pregnancy; patient was in amenorrhea after the procedure, and both an ultrasound scan and a diagnostic hysteroscopy revealed a retained product of conception. C. 40-year-old primiparous woman with a placental remnant of 30 mm after a miscarriage at 12 weeks. She had AUB (Abnormal uterine bleeding).

The mean size of placental remnants was reported to be 2cm (range 15 to 30 mm) in 49 patients (94%) by ultrasound imaging (Figure 1). Placental remnants filled the whole uterine cavity in 3 cases. Hysteroscopic resection of placental remnants required a median time of 4.5 minutes (range; 3 – 8 minutes) with a total procedure median time of 6.6 minutes (range: 5 - 10 minutes). In our cases, the median inflow fluid balance was 1380 ml (range: 700-4000), the median outflow 1200 ml (range: 500-3500), and the median deficit 240 ml (range: 100-400). Surgery outcomes are shown in Table III.

Intra-operative bleeding occurred in 3 cases. In 2 of them the placental remnants filled the whole cavity. For the first case we opted for a “shark jaw” blade offering a deeper grasp in order to successfully remove all the placental remnants. Whereas in the second case, the surgeon decided to alternate to bipolar resectoscopy. Finally, in the third case, where placental remnant obscured the whole cavity, RPOCs were first reduced by means of a grasping forceps and then removed with the IBS ® . No second-look surgery was considered necessary, as the uterine cavity was completely free of placental remnants at the end of the procedure (Figure 1). Patients were kept under observation in the hospital for 6 hours after the procedure and then discharged on the same day, with follow up instructions at 5-6 weeks.

After the first post-operative menstrual period, all patients underwent a transvaginal ultrasound scan to assess the endometrial cavity and to confirm the absence of any placental remnants. In all cases, ultrasound confirmed a smooth endometrium. In only one patient minor adhesions were found after 3D vaginal ultrasound.

## Discussion

Delayed bleeding with, or without, fever could be caused by RPOC after miscarriage. In most cases there is no clinical suspicion of retained placental tissue prior to the onset of symptoms and only in few cases amenorrhea may occur. Similarly, to what has been reported in the literature, in the present study, patients experienced the following clinical symptoms: 61% abnormal uterine bleeding, 29% asymptomatic, 10% subfertility and amenorrhea.

In regard to diagnosis, ultrasound is the best tool to diagnose RPOC (Hooker et al., 2014). Its most accurate feature (positive predictive value 80%) is the presence of an echogenic focus allowing to observe a thickened endometrium, if more than 10mm (Abbasi et al., 2008).

In 90% of our patients (47/52), RPOC was determined by transvaginal ultrasound. In most of these (80%), RPOC occurred after miscarriage or TOP (10%), whereas in the remaining 10% placental remnants occurred after delivery (by both vaginal and caesarean section). If retained placental tissue is suspected, whether to proceed directly to surgical intervention, or try medical treatment remains controversial (Acharya et al., 2004).

In a majority of cases, spontaneous expulsion of the placental tissue occurs within 2–4 weeks (Hooker et al., 2011). However, when conventional methods have been exhausted surgery is the only option. Another question that must be addressed is the choice between curettage and hysteroscopy, as primary surgical intervention. Traditionally D&C has been the first choice surgery, however this trend has changed with operative hysteroscopy.

One in five women encounter IUAs after miscarriage. In more than half of these cases, the extent of these adhesions is mild and without clinical relevance. Recurrent miscarriages and D&C procedures were identified as key risk factors for adhesion formation. The risk of adhesion increased up to 40% if repeated D&C is performed (Hooker et al., 2016). Therefore it is reasonable to assume that direct visualization offered by hysteroscopy reduces the risk of uterine perforation. Plus, the risk of incomplete removal of retained placenta will also be decreased, certainly leading to fewer IUA’s. Hysteroscopic removal of placental remnants, has been registered by several authors as an alternative method to ‘blind’ aspiration and/or curettage (Goldenberg et al., 1997; Westendorp et al., 1998; Golan et al., 2011; Hooker et al., 2013; Tjalina et al., 2013). The advantages of this approach are well established. Direct visualization of the RPOC, complete removal without damaging the surrounding healthy endometrium, and availability of a specimen for pathologic analysis make this approach preferable to ‘blind’ D&C.

Goldenberg et al. (1997) reported their experiences with 18 patients (16 post-abortion and 2 postpartum) who underwent a hysteroscopy for removal of residual trophoblastic tissue causing continuous bleeding. Complete removal of the residual tissue was achieved in all these patients with no short-term complications reported. The authors concluded that selective curettage of residual trophoblastic tissue performed under hysteroscopy was a safe and fast procedure, and could be the first-choice procedure, compared to conventional non-selective, ‘blind’ curettage. However, the current low level of surgeons’ preference for hysteroscopy, versus the traditional D&C for RPOC, reflects some disadvantages with the current approach. Some of these are listed below:

1. Resectoscopy requires skill and has a long learning curve.
2. Abundant bleeding may impair the sight, making the procedure very difficult. In such cases a good irrigation system is crucial to maintain a clear sight of the uterine cavity.
3. In some cases, the resectoscope loop may bend.
4. Before the introduction of the bipolar resectoscope, the concern of fluid overload and water intoxication with Hypernatremia must be considered.

In order to increase surgeon adoption of hysteroscopy for RPOC, the new Shaver technique address these challenges. Specifically, with the IBS ® method the following advantages are observed:

- A good visualization of the uterine cavity and the relative intrauterine pathology, through the removal of tissue chips at the same time as the resection. When the resectoscope is used, tissue chips may impair vision. This requires the surgeon to perform repeated in-and-out movements, with a higher risk of perforation and cervical laceration. Once inserted into the uterine cavity, the IBS ® is left in place for the entire duration of the procedure.
- A reduced risk of intravasation due to the use of saline solution, the brevity of the procedure, and a very low fluid deficit. In our study, a fluid loss limit of 2500 mL was reached in 1 patient only. In addition, no fluid-related adverse events were observed.
- A much more precise and clean surgery, due to the good visualization and direct action of the IBS ® over the pathological tissue. Moreover, the gentle mechanical technique and the blunt tip of the IBS ® , limits the damage to the healthy surrounding endometrium, leading to a reduced risk of uterine perforation, formation of intrauterine adhesions and Asherman Syndrome (Al-Inany et al., 2001; Hooker et al., 2011; Hamerlynck et al., 2013).
- A narrower outer instrument sheath of 8.5 mm (24 Fr.) allows for cervical dilatation only up to 8 of Hegar, instead of 9.5 of Hegar (of the 26 Fr. resectoscope). This leads to a reduced cervical trauma, with a lower risk of uterine perforation. In our cases, no uterine perforation occurred. Interestingly, a 19 Fr. IBS ® has been developed and is available, which should further improve these results.
- The possibility to collect all tissues for pathologic examination.
- The convenience of the re-usability of the shaver
- A very short learning curve for the IBS ® procedure may encourage gynaecologists to choose operative hysteroscopy instead of D&C for removing RPOC.

(To note: the longer the interval between the end of pregnancy and the operative hysteroscopy with IBS ® , the easier the procedure. As reported in the literature (Golan et al., 2011), devascularization (time related) of retained placenta may lead to less bleeding)

However, one limitation of the IBS ® is the impossibility of coagulation during the operation. This must taken into account in cases of heavy bleeding. Nevertheless, if necessary, a bipolar probe to selectively coagulate vessels can be introduced through the straight operative channel of the optics. In the presented cases, despite the lack of coagulation, no postoperative bleeding was observed.

Our data confirm precedent studies (Golan et al; 2011; Hamerlynck et al., 2013) which suggest that to prevent intraoperative bleeding and further reduce the risk of incomplete removal of placental remnants, an await of at least 2 to 3 months after pregnancy before hysteroscopic removal of placental remnants should be respected. To our knowledge, this is the first study on the removal of placental remnants with the IBS ® (Bigatti, 2011). It should also be noted that a number of studies have shown good results for the use of a morcellator to remove placental remnants (Pather et al., 2005). Furthermore, our results are similar to those reported in the literature on hysteroscopic surgery, including both resectoscope and morcellator to treat RPOC (Hamerlynck et al., 2013).

We had no postoperative complications, no need for second-look surgery, and had complete removal of RPOC after one hysteroscopic surgery, with satisfactory outcomes at the 6-week follow-up. This study confirms the already well-known advantages of the IBS and specifically it’s use for the removal of placental remnants (Bigatti, 2011).

## Conclusion

This is the first study using the hysteroscopic IBS ® for the removal of placental remnants.

This technique has shown to be an effective treatment method in the management of RPOC. We did not experience any complications, such as uterine perforation. In our study, we found that the longer the surgical procedure was performed after miscarriage, the less bleeding was observed. This is certainly due to the devascularization of the placental remnants which is time related. Thus, a reduced bleeding, allowed the surgeon to finalize the procedure in a single step. Adverse events, such as intrauterine bleeding may occur when using this technique, especially when treating large placental remnants. In the study, IBS ® demonstrated a good outcome in terms of operative time, fluid deficit, ease of procedure and postoperative adhesion. It is a safe, reliable, cost-effective and reusable tool for removing placental remnants. These results, as well as other outcomes, should however be confirmed by further studies with larger patient samples. More data is required for the preoperative decision over which modality is the safest and most complete to remove placental remnants for each patient. A randomized clinical trial with a larger number of patients should be therefore planned to determine which is the best modality for removal of placental remnants.

## Declaration of interests:

The authors report no declarations of interest and confirm that they have obtained the written permission from all patients whose case is being presented.
